# The role of TEAD4 in trophectoderm commitment and development is not conserved in non-rodent mammals

**DOI:** 10.1242/dev.202993

**Published:** 2024-09-24

**Authors:** Alba Pérez-Gómez, Leopoldo González-Brusi, Inés Flores-Borobia, Beatriz Galiano-Cogolludo, Ismael Lamas-Toranzo, Julieta G. Hamze, Adolfo Toledano-Díaz, Julián Santiago-Moreno, Priscila Ramos-Ibeas, Pablo Bermejo-Álvarez

**Affiliations:** Animal Reproduction Department, INIA, CSIC, Madrid 28404, Spain

**Keywords:** Bovine, Human, Rabbit, TEAD4 KO, Trophectoderm

## Abstract

The first lineage differentiation in mammals gives rise to the inner cell mass and the trophectoderm (TE). In mice, TEAD4 is a master regulator of TE commitment, as it regulates the expression of other TE-specific genes and its ablation prevents blastocyst formation, but its role in other mammals remains unclear. Herein, we have observed that *TEAD4* ablation in two phylogenetically distant species (bovine and rabbit) does not impede TE differentiation, blastocyst formation and the expression of TE markers, such as GATA3 and CDX2, although a reduced number of cells in the inner cell mass was observed in bovine *TEAD4* knockout (KO) blastocysts. Transcriptional analysis in bovine blastocysts revealed no major transcriptional effect of the ablation, although the expression of hypoblast and Hippo signalling-related genes tended to be decreased in KO embryos. Experiments were conducted in the bovine model to determine whether *TEAD4* was required for post-hatching development. *TEAD4* KO spherical conceptuses showed normal development of the embryonic disc and TE, but hypoblast migration rate was reduced. At later stages of development (tubular conceptuses), no differences were observed between KO and wild-type conceptuses.

## Teaser

**Summary:** TEAD4, a master regulator of trophectoderm differentiation in mice, was found to be dispensable for trophectoderm development in bovine and rabbit.

## INTRODUCTION

Early mammalian development relies on a series of cell differentiation events that give rise to specific embryonic lineages. The first lineage differentiation occurs at the morula stage and differentiates inner and outer cells into the inner cell mass (ICM) and the trophectoderm (TE), respectively (Berg et al., 2011). Cell differentiation confers lineage-specific functional properties that result in the formation of an inner cavity termed the blastocoel (Kim and Bedzhov, 2022), which characterizes the blastocyst stage. As a consequence of TE growth and expansion, the embryo hatches from a glycoprotein layer termed the zona pellucida and, around that time, a second differentiation event in the ICM gives rise to the hypoblast or primitive endoderm (a second extra-embryonic lineage), and the epiblast, from which the embryo proper will derive (reviewed by Pérez-Gómez et al., 2021a).

The molecular regulation of the first lineage differentiation has been thoroughly studied by means of knockout (KO) mouse models. However, the knowledge gathered from mouse models may not be applicable to other mammals, as remarkable differences in the molecular regulation of embryo development have been observed between mice and other mammals. For instance, the transcription factor (TF) EOMES, which determines the formation of TE and mesoderm in mice (Russ et al., 2000), is not expressed by the bovine TE (Berg et al., 2011). Similarly, whereas CDX2 is exclusively expressed by the TE in mice and other mammals (Pérez-Gómez et al., 2021a), *CDX2* downregulation (Goissis and Cibelli, 2014) or ablation (Shi et al., 2023; A.P.-G., L.G.-B., I.F.-B., B.G.-C., J.G.H., P.R.-I. and P.B.-A., unpublished) in bovine embryos does not disrupt blastocyst formation. This contrasts with the phenotype of *Cdx2* KO mice embryos, which are unable to maintain their blastocoels as a result of defects in TE epithelial integrity (Strumpf et al., 2005). The lack of tools to generate KO models in mammals other than mice has hampered the study of this process in other species, but the development of genome-editing tools now allows gene ablation in other mammals (Lamas-Toranzo et al., 2017).

Acting upstream of other TE-specific TFs, the Hippo signalling pathway plays a central role in murine TE fate determination (Nishioka et al., 2009), which is apparently conserved across different mammals (Gerri et al., 2020). The core components of the Hippo pathway are the protein kinases MST1/2 and LATS1/2, their respective co-factors SAV1 and MOB1, the transcriptional co-activators YAP1 and TAZ (WWTR1), and the transcription factors TEAD1-4 (Zhao et al., 2010). TEAD factors are the ultimate effectors of Hippo signalling pathway, being responsible for the regulation of downstream transcription factors. Of the four TEAD family members, TEAD4 is the only member deemed essential for TE specification in mouse embryos, acting at a level upstream of other TE-specific transcription factors: *Tead4* ablation prevents mouse blastocyst formation (Nishioka et al., 2009; Yagi et al., 2007) by impeding the expression of downstream genes, such as *Cdx2*, *Eomes* or *Gata3* (Nishioka et al., 2008; Yagi et al., 2007; Ralston et al., 2010). However, gene-ablation experiments in bovine (Pérez-Gómez et al., 2021b; Wu et al., 2024) and human (Stamatiadis et al., 2022) embryos have shown that *TEAD4* is dispensable for blastocyst formation. A plausible explanation for these discrepancies may be that TEAD4 is required for later stages of TE differentiation/proliferation, rather than initial TE specification, which would be in line with the earlier timeline of other developmental events (e.g. X-chromosome inactivation; Bermejo-Alvarez et al., 2010b; Okamoto et al., 2011) observed in mouse embryos compared with other mammals.

The objective of this study was to determine the role of TEAD4 in TE specification and maintenance in non-rodent species (bovine and rabbits). Ungulate embryos constitute an interesting model in which to study TE specification and maintenance as, following hatching, they undergo a prolonged period of pre-implantation development termed conceptus elongation when extra-embryonic membranes (TE and hypoblast) proliferate extensively without direct interactions with maternal cells. An experiment was also conducted in rabbits – a non-rodent and non-ungulate model – to determine whether TEAD4 is dispensable for TE commitment and blastocyst formation in a phylogenetically distant species.

## RESULTS

### TEAD4 expression during bovine preimplantation development

**Fig. 1.**
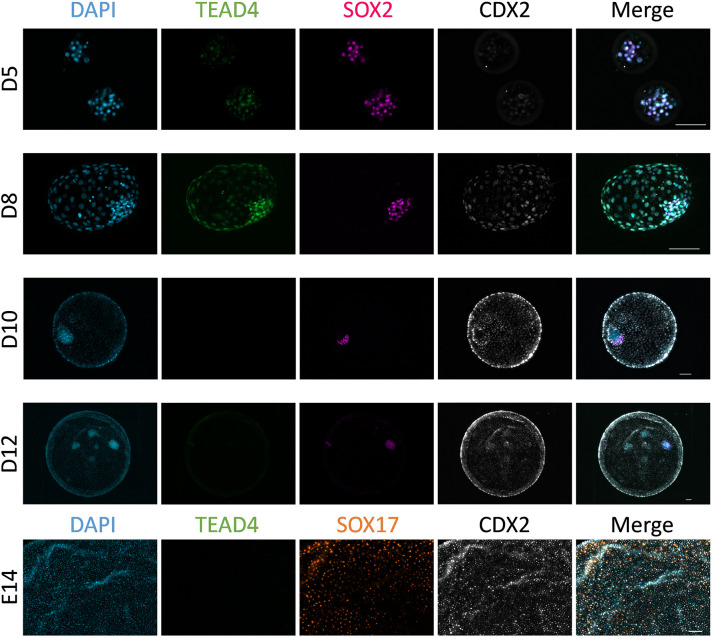
**Expression pattern of TEAD4 in bovine embryos from morula to tubular stages.** Representative images (ten embryos analyzed per stage) of D5 morula, D8 blastocyst, D10 and D12 embryos and extra-embryonic membranes from E14 conceptus. IHC analysis for TEAD4, CDX2 (trophectoderm) and SOX2 (inner cell mass/epiblast) or SOX17 (hypoblast), with cell nuclei counterstained with DAPI. Scale bars: 100 µm.

In order to determine the expression pattern of TEAD4 during bovine preimplantation development, TEAD4 expression was analyzed by immunohistochemistry (IHC). TEAD4 and the ICM-specific TF SOX2 were located in the nucleus in both inner and outer cells of the morula [days post-*in vitro* fertilization (D) 5], whereas the TE-specific TF CDX2 was only starting to be weakly expressed in the outer cells at this time point (Fig. 1). At the expanded blastocyst stage (D8), clear ICM/TE differentiation was apparent, given the ICM- and TE-exclusive expression of SOX2 and CDX2, respectively, but TEAD4 signal was detected in the nuclei of both ICM and TE cells. Following blastocyst hatching and prior to hypoblast migration (D10), TEAD4 signal was not detected and remained undetectable at later stages: spherical conceptuses showing hypoblast migration (D12) and extra-embryonic membranes (formed by hypoblast and trophectoderm) of tubular conceptuses [embryonic day (E) 14].

### *TEAD4* KO embryonic development to blastocyst

**Fig. 2.**
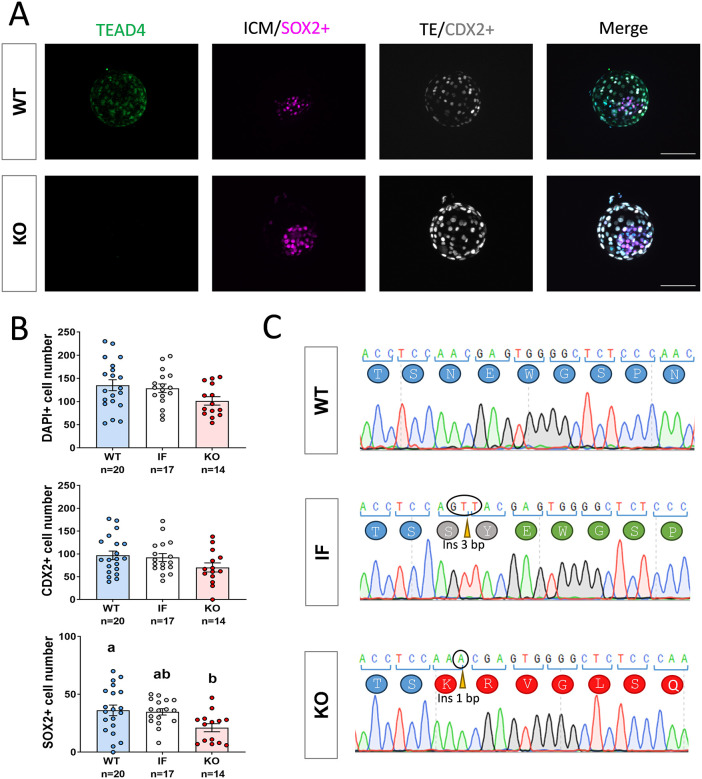
**Developmental analysis of *TEAD4* KO bovine blastocysts.** (A) Representative images (number of embryos analyzed shown in B) of WT and *TEAD4* KO D8 blastocysts subjected to IHC for TEAD4, SOX2 (inner cell mass) and CDX2 (trophectoderm). Scale bars: 100 µm. (B) Graphical representation (mean±s.e.m.) of DAPI^+^ (total), CDX2^+^ (trophectoderm) and SOX2^+^ (inner cell mass) cell numbers in WT, edited in-frame (IF) and KO D8 blastocysts. Different letters indicate significant differences based on ANOVA *P*<0.05. (C) Sanger sequencing chromatograms of WT, IF and KO alleles with their corresponding amino acid sequence. IF alleles are composed of indels that are a multiple of three (3 bp insertion in the example) that do not disrupt the open reading frame. In contrast, KO alleles are formed by frame-disrupting indels (1 bp insertion in the example). KO embryos harbour only KO alleles.

The similar localization pattern of TEAD4 in both ICM and TE observed by IHC suggests that it may be dispensable for ICM/TE differentiation. To assess unequivocally the role of TEAD4 during the first lineage differentiation, the development of *TEAD4* KO embryos was assessed *in vitro*. TEAD4 ablation in bovine embryos did not disrupt blastocyst formation, as similar blastocyst formation rates were obtained for wild-type (WT) embryos injected with *Cas9* alone (C group) and embryos injected with *Cas9* mRNA and single guide RNA (sgRNA) against *TEAD4* (C+G group; partly composed of KO embryos) (Table 1). Editing efficiency in the C+G group was ∼94% (31/33) and KO generation efficiency (i.e. the percentage of embryos containing only KO alleles formed by frame-disrupting indels) was 45% (14/31). As expected, KO embryos did not show a TEAD4 signal by IHC (Fig. 2A). *TEAD4* KO embryos displayed an overtly normal morphology and the number of total and TE (CDX2^+^) cells was similar in KO, edited in-frame (IF) and WT embryos (Fig. 2B). However, a small but statistically significant reduction in the number of ICM (SOX2^+^) cells was observed for KO embryos compared with their WT counterparts (Fig. 2B). IF embryos are edited embryos containing at least one allele in which the open reading frame of the gene is conserved, often containing an edited allele composed of an IF indel causing a minor change in TEAD4 protein sequence such as that shown in Fig. 2C.

**Table 1.**
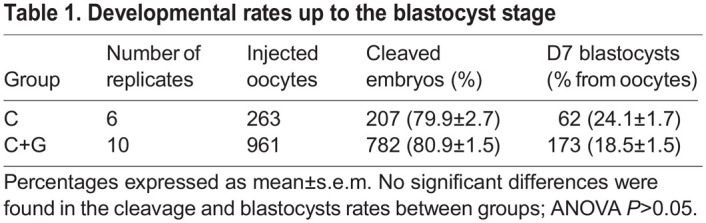
Developmental rates up to the blastocyst stage

### Transcriptional analysis of *TEAD4* KO blastocysts

**Fig. 3.**
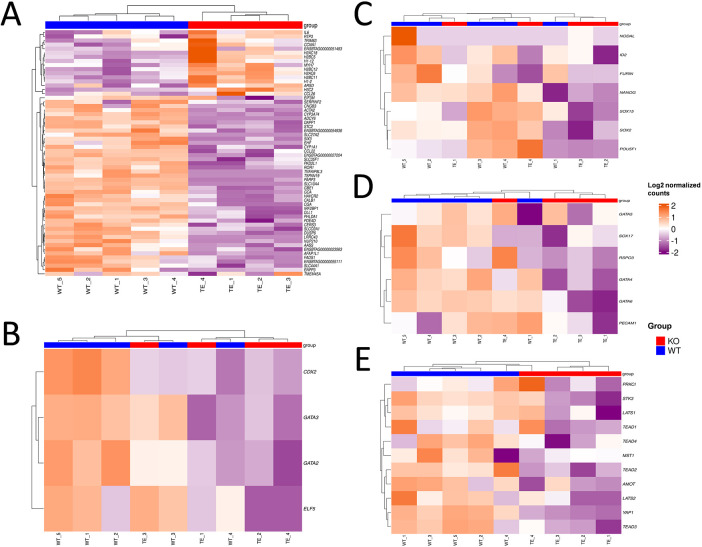
**Transcriptional analysis of WT and *TEAD4* KO D8 blastocysts.** (A-E) Hierarchical clustering and heat maps based on the differentially expressed genes at an adjusted *P*-value <0.05 and a shrunken fold change >2 (A), trophectoderm-related genes (B), epiblast-related genes (C), hypoblast-related genes (D) and Hippo signalling-related genes (E).

In the absence of a noticeable effect of *TEAD4* ablation on TE or ICM differentiation and proliferation, a transcriptional analysis was conducted to determine possible transcriptional alterations induced by the absence of TEAD4. Transcriptional analysis of *TEAD4* KO blastocysts detected 14,598 genes considering a median expression across samples over the threshold of 0.1 transcripts per million. Considering a *P*-adjusted value of 0.05 and a shrunken fold change >2, 64 genes were differentially expressed: 18 upregulated and 46 downregulated in KO blastocysts compared with their WT counterparts (Fig. 3A, Table S1). Tailored hierarchical clustering based on the expression of trophectoderm-, hypoblast-, epiblast- and Hippo signalling-related genes was conducted to determine the effect of *TEAD4* ablation on the expression of TFs involved in lineage specification and on Hippo signalling genes (Fig. 3B-E). Hierarchical clustering based on the expression of key TFs for trophectoderm or epiblast failed to group the samples according to genotype. However, when samples were clustered according to the expression of hypoblast-related genes they tended to group according to genotype (only one KO sample clustered with WT samples; Fig. 3D), and when they were clustered according to the expression of Hippo signalling genes they grouped according to genotype (Fig. 3E).

### Assessment of later stages of *TEAD4* KO development

**Fig. 4.**
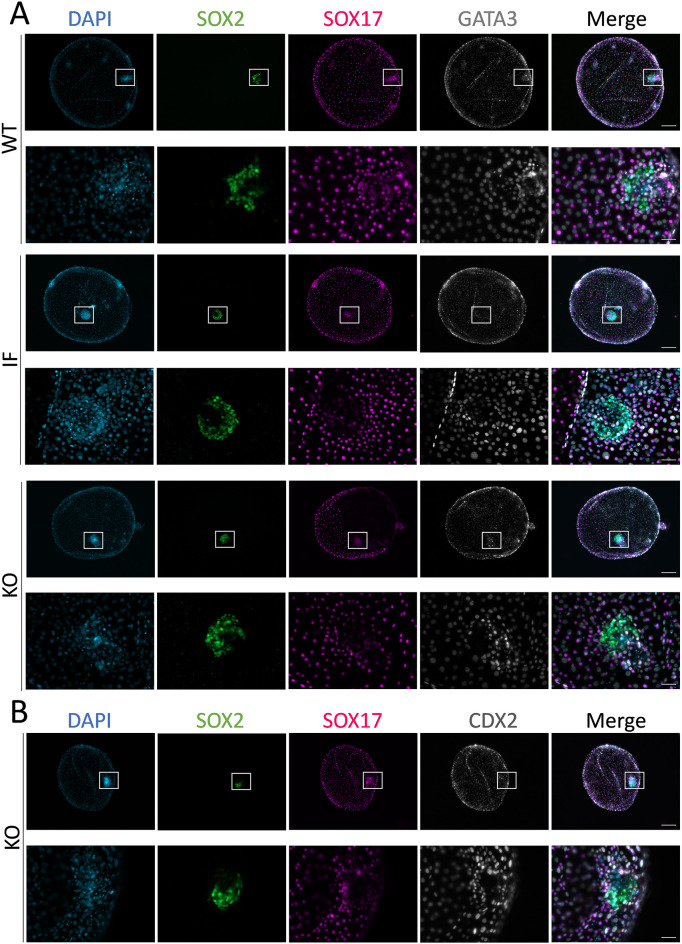
**Developmental analysis of *TEAD4* KO spherical bovine D12 conceptuses.** (A) Representative images (29 WT, 22 IF and 23 KO) of WT, IF and *TEAD4* KO D12 conceptuses subjected to IHC for SOX2 (epiblast), SOX17 (hypoblast) and GATA3 (trophectoderm). (B) Representative images of a D12 *TEAD4* KO embryo showing normal development of the epiblast (SOX2^+^), proliferation of the hypoblast (SOX17^+^) and expressing CDX2 in the trophectoderm. Nuclei were counterstained with DAPI. White squares in upper images showing whole conceptuses denote the limits of the magnification of the EDs shown in the panels below. Scale bars: 200 µm (conceptus images); 50 µm (ED images).

In order to determine whether *TEAD4* ablation impaired embryo development at further stages of preimplantation development, the development of *TEAD4* KO embryos was assessed up to D12 *in vitro* or to E14 *in vivo*. The development of bovine embryos to early post-hatching stages was not disrupted by TEAD4 ablation. No significant differences in survival rates from D7 to D12 were observed between C+G and C groups (Table 2). All D12 embryos analysed in the C+G group were edited and ∼51% (23/45) were KO. The development of TE cells was not affected by TEAD4 ablation, as D12 KO embryos were similar in size to WT and IF embryos and expressed the TE markers CDX2 and GATA3 (Fig. 4). Epiblast development was not affected by TEAD4 ablation either, as epiblast survival and embryonic disc (ED) formation rates and the number of epiblast cells (SOX2^+^) were similar between KO, IF and WT embryos. However, although hypoblast differentiation was achieved in *TEAD4* KO embryos, as evidenced by the presence of SOX17^+^ cells, hypoblast migration rates were lower in KO embryos compared with WT and IF embryos (Table 3).

**Table 2.**
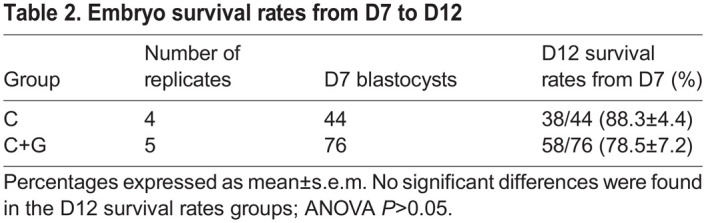
Embryo survival rates from D7 to D12

**Table 3.**
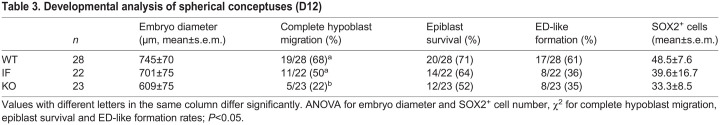
Developmental analysis of spherical conceptuses (D12)

**Fig. 5.**
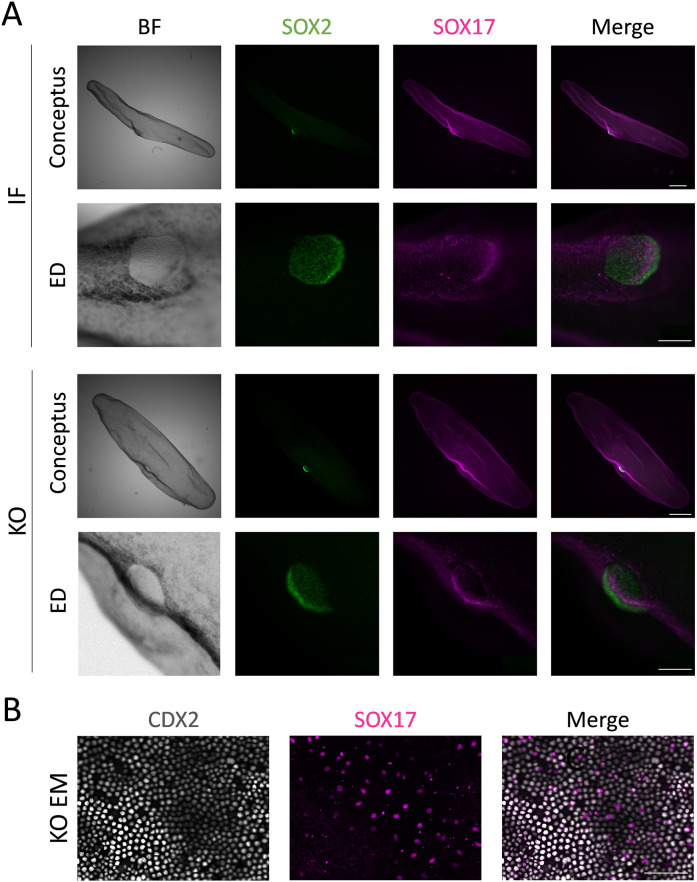
**Developmental analysis of *TEAD4* KO bovine tubular E14 conceptuses.** (A) Representative images (10 IF and 13 KO) of IF and KO conceptuses developing an ED and showing complete hypoblast migration. Bright-field (BF) and IHC images for SOX2 (epiblast) and SOX17 (hypoblast) cells of the whole conceptuses (upper images) or focusing into the ED (lower images). (B) Magnification of the extra-embryonic membranes of a *TEAD4* KO E14 conceptus showing expression of CDX2 in the trophectoderm layer, which is underlined by SOX17^+^ hypoblast cells. Scale bars: 1 mm (conceptus); 200 µm (ED); 100 µm (extra-embryonic membranes).

Conceptus development to tubular stages (E14) was not affected by TEAD4 ablation. Recovery rate following embryo transfer and uterine flushing was ∼42% (26/62), and edition and KO generation rates were ∼88% (23/26) and 50% (13/26), respectively. Conceptus length (indicative of the development of extra-embryonic membranes: TE and hypoblast) and hypoblast migration rates were comparable between KO, IF and KO embryos, and ED development – assessed by ED formation rate and diameter – was not affected either by TEAD4 ablation (Fig. 5, Table 4).

**Table 4.**
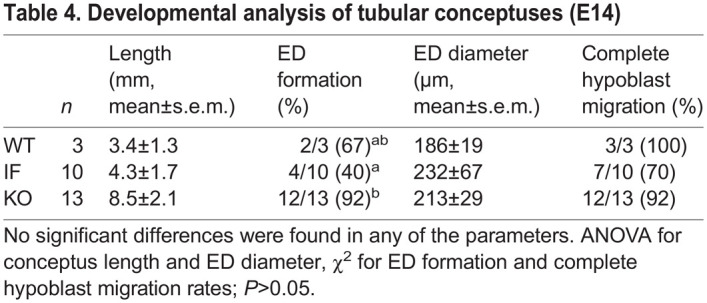
Developmental analysis of tubular conceptuses (E14)

### *TEAD4* KO in rabbit embryos

**Fig. 6.**
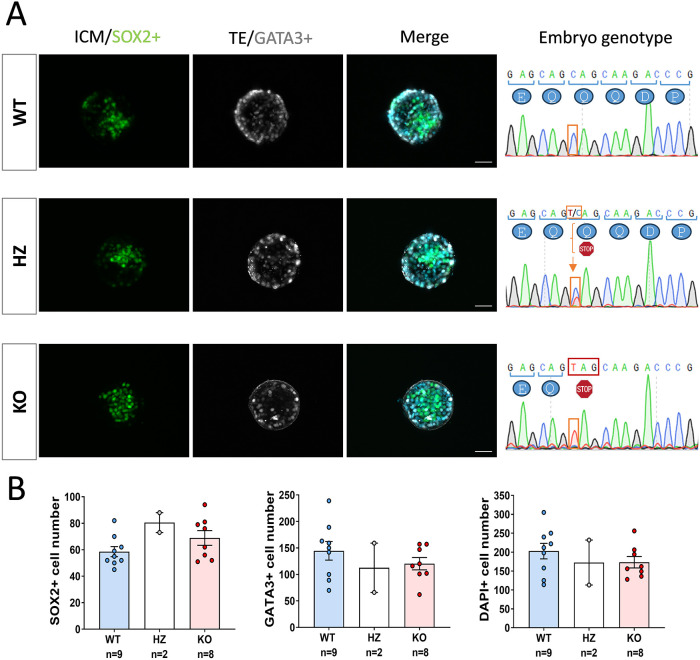
**Developmental analysis of *TEAD4* KO rabbit D5 blastocysts.** (A) Representative images (number of embryos analyzed shown in B) of WT, heterozygous (Hz) and KO blastocysts subjected to IHC to detect inner cell mass (SOX2) and trophectoderm (GATA3). Nuclei were counterstained with DAPI. Scale bars: 50 µm. Sanger sequencing chromatograms of each of the embryos are shown on the right. Gene ablation strategy consisted of achieving a C-T substitution by base editors, which convert a CAG codon into a TAG stop codon. Hz embryo shows the unconverted and converted versions (C and T) at the position marked with an orange arrow. (B) Graphical representation (mean±s.e.m.) of inner cell mass (SOX2^+^), trophectoderm (GATA3^+^) and total (DAPI^+^) cell numbers in WT, Hz and KO blastocysts. No significant differences were found between groups based on ANOVA (*P*>0.05).

To determine whether the essential role of TEAD4 for murine TE differentiation was conserved in a non-rodent species phylogenetically distant from ungulates, a gene-ablation experiment was conducted in rabbit embryos. Development to blastocyst was comparable between embryos injected with BE3-encoding mRNA and sgRNA against *TEAD4* (BE+G group; containing KO embryos, 10/12 zygotes developing to blastocyst) and embryos injected with BE3-encoding mRNA alone (BE group; only formed by WT embryos, 9/11 zygotes developing to blastocyst). All ten blastocysts from the BE+G group were edited and eight (80%) were KO, whereas the other two were heterozygous for WT and KO alleles. *TEAD4* KO blastocysts presented an overtly normal morphology, expressed the TE marker GATA3 and total inner cell mass (SOX2^+^) and trophectoderm (GATA3^+^) cell numbers were similar between KO and WT blastocysts (Fig. 6).

## DISCUSSION

The molecular regulation of cell differentiation events during early embryogenesis was initially assumed to be conserved between mice, the only mammalian species in which targeted genome modification could be achieved efficiently, and other mammals. However, gene-ablation experiments performed in different mammalian models and human embryos are uncovering an unexpected developmental diversity. Focusing on first lineage differentiation, crucial regulators of murine TE commitment, such as EOMES and CDX2, have been shown to be dispensable for TE differentiation in bovine embryos (Berg et al., 2011; Goissis and Cibelli, 2014; Shi et al., 2023), and the reciprocal exclusion of POU5F1 (OCT4) and CDX2 expression observed in the ICM and TE of the mouse blastocyst is not conserved in other mammals, such as bovine, rabbit, pig and human (Kirchhof et al., 2000; Kobolak et al., 2009; Hansis et al., 2000), owing to the lack of critical CDX2-responsive regulatory regions in *POU5F1* in non-rodent mammals (Berg et al., 2011; Kobolak et al., 2009). Acting upstream of other TE-specific TFs, such as CDX2, EOMES or GATA3 (Nishioka et al., 2008; Yagi et al., 2007; Ralston et al., 2010), TEAD4 may play a conserved role in mammalian TE differentiation by regulating these TE-specific TFs in mice or other unknown TFs required for TE commitment or proliferation in other mammals. However, the experiments conducted herein in two phylogenetically distant species (bovine: Artiodactyla; rabbit: Lagomorpha) indicate that the essential role of TEAD4 appears to be exclusive to rodents or mice, as the order Lagomorpha shares the same the same clade (Glires) as the order Rodentia (Alvarez-Carretero et al., 2022).

The dispensable role of TEAD4 for initial TE commitment in bovine and rabbit embryos agrees with prior gene-ablation studies in bovine and human embryos, which showed that *TEAD4* ablation does not prevent blastocyst formation (Pérez-Gómez et al., 2021b; Wu et al., 2024; Stamatiadis et al., 2022). However, the human study (Stamatiadis et al., 2022) revealed that, whereas *TEAD4* KO embryos developed to blastocysts and were able to express normally the TE marker GATA3, *TEAD4* ablation affected TE morphology and abolished the expression of CDX2 at that stage. These results suggested that, although initial TE commitment is achievable in the absence of TEAD4 in humans, TEAD4 may be required for subsequent TE differentiation or proliferation (i.e. beyond hatching), an extent that could not be proved in human embryos. To test whether *TEAD4* ablation prevented further TE development, we conducted experiments in bovine embryos following blastocyst hatching, revealing that TEAD4 is clearly dispensable for TE development as the TE developed normally in *TEAD4* KO conceptuses and the TEAD4 localization pattern was not compatible with a role in TE differentiation or proliferation: TEAD4 was localized in the nucleus in both the ICM and the TE, which has also been observed in human embryos (Stamatiadis et al., 2022), and was not detected at later developmental stages. The differences between bovine (Pérez-Gómez et al., 2021b; Wu et al., 2024; this paper) and human (Stamatiadis et al., 2022) studies may be species specific, but the dispensable role of *TEAD4* for TE differentiation observed in rabbits – a species phylogenetically closer to mice than humans (Alvarez-Carretero et al., 2022) and in which the ablation did not affect TE morphology – suggests that TEAD4 role is not conserved beyond the order Rodentia and thereby it could be also dispensable for human TE development. In this perspective, the altered TE morphology (reduced proliferation) and lack of expression of CDX2 observed in *TEAD4* KO human embryos may be a consequence of an overall developmental delay caused by *TEAD4* ablation.

The dispensable role of TEAD4 in TE commitment opens the question of how first lineage differentiation occurs in mammals other than mice. As the ablation of *CDX2* does not prevent TE differentiation in bovine (Shi et al., 2023; our unpublished observations) and the TE-specific TF GATA3 is also dispensable for TE commitment in bovine embryos (Shi et al., 2023), members of the Hippo pathway could substitute TEAD4 as a master regulator of initial TE commitment. Our RNA-sequencing (RNA-seq) analysis revealed that all four TEAD members were expressed by D8 blastocysts, but none of the other TEAD members were observed to be upregulated to compensate for the loss of TEAD4; indeed, all Hippo family members exhibited a non-significant reduction in their expression in KO embryos compared with WT. This finding suggests that Hippo signalling may be indeed dispensable for initial TE differentiation, but dedicated investigation would be required to determine whether this is the case.

In contrast to the normal TE development observed in the absence of TEAD4, *TEAD4* KO bovine embryos showed an unexpected reduction in the ICM cell number and hypoblast proliferation *in vitro* (D8 and D12, respectively), which was apparently corrected at later stages under *in vivo* conditions, where normal hypoblast proliferation was observed in most (12/13) of the E14 *TEAD4* KO conceptuses analysed. The altered hypoblast proliferation in *TEAD4* KO embryos at D12 seems to be linked to a reduction in hypoblast precursors at their less populated ICMs by D8. In agreement with this hypothesis, RNA-seq analysis observed an overall reduction in the expression of hypoblast-related genes in KO samples, which roughly grouped together in the hierarchical clustering conducted based on the expression of hypoblast-related genes, in contrast to the clusterings conducted based on the expression of epiblast or trophectoderm-related genes. RNA-seq also revealed that most of the upregulated genes in KO blastocysts (11/18) were histones (*H2BC11*, *H1-2*, *H2AC6*, *H2BC12*, *H3C2*, *H2BC5*, *H2AC18*, *H1-12* and *H2BC9*) and cyclins (*CCNA1* and *CCL26*), which may suggest a cellular response to increase a reduced cell proliferation. The upregulation of *IL6*, a cytokine activating the JAK-STAT pathway, which promotes hypoblast development in bovine blastocysts (Wooldridge and Ealy, 2021), may suggest a response to increase the number of hypoblast precursors. An alternative explanation for the transcriptional differences observed is that, similarly to what may have occurred in human KO embryos, *TEAD4* ablation may have simply caused a developmental delay in bovine embryos. A delayed development would be compatible with a reduced expression of hypoblast-differentiation genes (delayed second lineage differentiation) and a reduced proliferation of ICM and hypoblast cells at a fixed time point of observation that could be compensated for at later stages of development under optimal developmental conditions (*in vivo*). In agreement with this later possibility, *TEAD4* is known to be a promoter of cell proliferation in several cancers (e.g. Gu et al., 2020; Tang et al., 2018) by activating glycolytic metabolism (Zhan et al., 2023), which shares some similarities with that of the embryo (Krisher and Prather, 2012; Bermejo-Alvarez et al., 2010a).

In conclusion, the essential role of *Tead4* in first lineage commitment in mouse embryos is not conserved in other mammals: TEAD4 is dispensable for initial TE commitment and blastocyst formation in two phylogenetically distant species (bovine and rabbit) and is not required for TE development to advanced post-hatching stages in bovine conceptuses.

## MATERIALS AND METHODS

### Animals

Bovine experiments were conducted employing *in vitro*-produced embryos, which were developed to the blastocyst stage (D8) and early post-hatching stage (D12) *in vitro* or developed to a later post-hatching stage (E14) in the sheep uterus. Rabbit experimentation was conducted using *in vivo*-derived zygotes developed to the blastocyst stage (D5) *in vitro*. Animal experimentation was conducted following protocols approved by INIA Animal Care Committee and Madrid Region Authorities (protocols PROEX 040/17 and PROEX 059.2-22).

### Gene-ablation strategies and generation of CRISPR components

Bovine *TEAD4* ablation was achieved by the generation of randomly generated frame-disrupting insertion/deletion (indels) following non-homologous end joining repair (NHEJ) of the double-strand breaks (DSBs) generated by the conventional CRISPR/Cas9 system. The randomly generated indels disrupt the open reading frame of the gene when they are not multiple of three, leading to a truncated protein. An sgRNA targeting the fourth exon was designed using CRISPOR (Concordet and Haeussler, 2018) and synthesized and purified using the Guide-it sgRNA In Vitro Transcription Kit^®^ (Takara Bio) using a primer containing a T7 promoter and the sgRNA sequence (Table S2). Frame-disrupting indels resulted in proteins exhibiting a maximum homology of 22% (110 amino acid) with the WT protein in all predicted isoforms (XP_059743089.1 and XP_059743088.1). As alternative splicing and start sites have been reported to produce functional TEAD4 isoforms in human cells (NP_958849.1 and NP_958851.1; Qi et al., 2016; Rashidiani et al., 2024), a sequence alignment was conducted (BLAST, NCBI) to determine whether those functional proteins could be produced by the edited bovine transcripts (Fig. S1). The bovine proteins generated following frame disruption (i.e. KO alleles) would share a maximum homology with any human isoform of 10 aa and if the alternative start site generating the human isoform NP_958851.1 was in use in bovine embryos, the unexpected protein would be detected by the antibody ab151274 (Abcam; immunogen corresponding to residues 150-250 of NP_003204.2; https://abiomed.kz/product/anti-tead4-antibody-ab151274). Capped polyadenylated *Cas9* mRNA was *in vitro* transcribed with the mMESSAGE mMACHINE T7 ULTRA kit^®^ (Life Technologies) using the plasmid pMJ920 (Addgene #42234) linearized with BbsI and treated with Antarctic phosphatase (NEB) and purified using the MEGAClear kit (Life Technologies) (Bermejo-Alvarez et al., 2015).

Rabbit *TEAD4* ablation was achieved by the generation of a stop codon by the cytosine base editor BE3 (CBE) (Komor et al., 2016). Instead of generating a DSB that will result in randomly generated indels, CBE converts cytosine to thymidine, a conversion that can generate a stop codon and thereby disrupt protein formation. An sgRNA was designed at the eighth exon to convert a CAG codon into a TAG stop codons and truncate the protein to 162 amino acids (45% protein length) in all predicted isoforms (XP_017198872.1, XP_051706193.1 and XP_051706192.1). As alternative splicing and start sites have been reported to produce functional TEAD4 isoforms in human cells (NP_958849.1 and NP_958851.1; Qi et al., 2016; Rashidiani et al., 2024), a sequence alignment was conducted (BLAST, NCBI) to determine whether those functional proteins could be produced by the edited rabbit transcript (Fig. S1). The protein generated by the rabbit KO allele shares homology with diverse ranges of the human isoforms (residues 73-235 for NP_003204.2, 73-192 for NP_958849.1 and 1-106 for NP_958851.1), which roughly correspond to ∼30-37% of the human proteins and in all cases, the stop codon is generated downstream of the start codon, so no alternative shorter functional proteins are expected. The sgRNA was produced as described above using a specific primer for the target sequence (Table S2). Capped polyadenlyated BE3-encoding mRNA was produced by *in vitro* transcription using the mMESSAGE mMACHINE T7 ULTRA kit^®^ (Life Technologies) and the plasmid pCMV-BE3 (Addgene #73021) linearized with BbsI. mRNA was purified using the MEGAClear kit (Life Technologies).

### *In vitro* production of bovine *TEAD4* KO embryos

Genome-edited bovine embryos were produced *in vitro* following protocols previously described (Lamas-Toranzo et al., 2019b). Briefly, immature cumulus–oocyte complexes (COCs) were obtained by aspirating 2-8 mm follicles from bovine ovaries collected at local slaughterhouses. COCs were selected based on conventional morphological criteria and matured for 24 h in groups of 50 in four-well dishes containing TCM-199 medium supplemented with 10% (v/v) fetal calf serum (FCS) and 10 ng/ml epidermal growth factor at 38.5°C and 5% CO_2_ in the air with humidified atmosphere. Matured oocytes were denuded by vortexing for 3 min in 1 ml of 300 µg/ml hyaluronidase and randomly divided into two groups for microinjection. One group was microinjected with a solution of *Cas9* mRNA and sgRNA against *TEAD4* (C+G group) at a concentration of 300 and 100 ng/µl, respectively, and another with *Cas9* alone at 300 ng/µl, serving as an injection control (C group). Cytoplasmic microinjection was performed on denuded oocytes using a filament needle under a Leica DMi8 inverted microscope assisted by Femtojet (Eppendorf).

Immediately following microinjection, *in vitro* fertilization was carried out with frozen–thawed spermatozoa from a single stud bull selected through a gradient of 40-80% Bovipure (Nidacon Laboratories AB). Spermatozoa were co-incubated with 30-50 microinjected oocytes at a final concentration of 10^6^ spermatozoa/ml in TALP medium supplemented with 10 mg/ml heparin, in four-well plates at 38.5°C in an atmosphere of 5% CO_2_ and maximum humidity. At approximately 20 h post-insemination (hpi), presumptive zygotes were vortexed for 30 s to remove the spermatozoa attached to the zona pellucida and cultured in groups of 20-25 in 25 μl droplets of synthetic oviduct fluid (SOF) (Holm et al., 1999), supplemented with 5% FCS under mineral oil. Culture took place at 38.5°C in an atmosphere of 5% CO_2_, 5% O_2_ and 90% N_2_ with maximum humidity. Cleavage and blastocysts rates were evaluated at 48 hpi and 7 days post-insemination. For IHC analysis, zona-free D8 blastocysts were fixed in 4% paraformaldehyde for 15 min at room temperature (RT) and stored in PBS with 1% bovine serum albumin (BSA) until analysis. For transcriptomic analysis, D8 blastocysts were snap-frozen at the bottom of a cryotube and stored at −80°C.

Post-hatching culture was carried out following an optimized protocol described by Ramos-Ibeas et al. (2023). Briefly, D7 blastocysts were cultured in 500 μl of N2B27 medium (1:1 Neurobasal and DMEM/F12 medium supplemented with 1× penicillin/streptomycin, 2 mM glutamine, 1× N2 and 1× B27 supplements; Thermo Fisher Scientific). Post-hatching culture took place at 38.5°C in a water-saturated atmosphere of 5% CO_2_, 5% O_2_ and 90% N_2_ and half of the culture medium was replaced every second day. Embryos remained in culture until D12, when embryo survival was analysed (alive embryos were able to maintain the blastocoel, whereas dead embryos collapsed). Surviving embryos were fixed as described above. To evaluate TEAD4 expression during preimplantation development, non-injected embryos were collected at D5 (18 embryos), D8 (12 embryos), D10 (11 embryos) and D12 (23 embryos) and fixed as described above.

### *In vivo* development of bovine embryos to E14

To assess the developmental ability of KO embryos to reach stages of development that cannot be currently achieved by *in vitro* systems, D7 blastocysts from the C+G group (i.e. partially composed of KO embryos) were transferred to two recipient 2- to 3-year-old ewes. Oestrous synchronization was achieved by the insertion of a progesterone-releasing device (CIDR-Ovis, Zoetis). 100 µg of cloprostenol (Estrumate^®^, MSD) were administered 11 days after CIDR insertion and 450 IU of PMSG (Sincropart PMSG, CEVA) were administered on the day of CIDR removal (12 days after insertion). Embryo transfer took place 8 days after CIDR removal. The presence of corpora lutea was assessed by abdominal laparoscopy (both recipients showed one corpus luteum in each ovary) and the cranial section of the uterine horns were exteriorized to introduce 15-16 bovine blastocysts from C+G groups per uterine horn. Following embryo transfer, corpora lutea were maintained by CIDR insertion and embryos were recovered 9 days after embryo transfer at a developmental stage equivalent to E14 by post-mortem (Rexroad and Powell, 1999) flushing of the uterine horns with Euroflush® (IMV). Collected conceptuses were measured and fixed as described above.

### Generation of rabbit *TEAD4* KO embryos

Gene-edited rabbit embryos were generated following protocols similar to those described by Lamas-Toranzo et al. (2019a). Rabbit zygotes were recovered post-mortem from 6- to 15-month-old female rabbits by oviductal flushing at 14 h after artificial insemination and induction of ovulation by the administration of 0.02 mg of gonadorelin (Inducel, Laboratorios Ovejero). Experiments were conducted in two independent replicates, each corresponding to a female. Immediately after collection, zygotes were randomly divided in two groups for microinjection. One group was microinjected with a solution of BE3-encoding mRNA and sgRNA against *TEAD4* (BE+G group) at a concentration of 200 and 100 ng/µl, respectively, and another with BE3-encoding mRNA alone at 200 ng/µl, serving as an injection control (BE group). Cytoplasmic microinjection was performed using a filament needle under a Leica DMi8 inverted microscope assisted by Femtojet (Eppendorf). Following microinjection, zygotes were cultured in 25 μl droplets of TCM-199 medium supplemented with 5% FCS under mineral oil. Culture took place at 38.5°C in an atmosphere of 5% CO_2_, 5% O_2_ and 90% N_2_ with maximum humidity. Cleavage and blastocysts rates were evaluated at 42 and 116 (D5) hpi. D5 blastocysts were fixed in 4% paraformaldehyde for 15 min at RT and stored in PBS until analysis.

### Lineage development analysis by IHC

D8, D12 and E14 bovine embryos and D5 rabbit blastocysts fixed as described above were washed in PBS-1% BSA and permeabilized in 1% Triton X-100 in PBS for 15 min at RT and blocked in 10% FCS-0.02% Tween 20 in PBS for 1 h at RT. Subsequently, specimens were incubated overnight at 4°C with primary antibodies (CDX2, Biogenex MU392A-UC; GATA3, Abcam ab199428; SOX17, R&D AF1924; SOX2, Invitrogen 14-9811-80) at 1:100. After four washes in PBS-1% BSA, embryos were incubated in a solution of DAPI and the appropriate secondary Alexa-conjugated antibodies (donkey anti-rat IgG Alexa Fluor^TM^ 488, donkey anti-goat IgG Alexa Fluor^TM^ 555 and donkey anti-mouse IgG Alexa Fluor^TM^ 647; Life Technologies) at 1:300 for 1 h at RT, followed by four washes in PBS-1% BSA.

For TEAD4 detection, immunohistochemistry protocol was adapted from a previously published method (Sakurai et al., 2017). Fixed specimens were permeabilized in 0.3% Triton X-100 in PBS for 60 min and washed twice in 0.1% Triton X-100 in PBS (TXPBS) for 10 min. Blocking was performed by incubation in 0.5% BSA-10% FCS in TXPBS for 90 min at RT. Embryos were incubated with anti-TEAD4 primary antibody (Abcam ab151274, 1:100) in 0.5% BSA – 0.05% Triton X-100 in PBS for 2 h at RT. Then, embryos were washed four times in TXPBS and transferred to a solution with the appropriate secondary Alexa-conjugated antibody (1:300; donkey anti-rabbit IgG Alexa Fluor^TM^ 488) in 0.5% BSA-0.005% Triton X-100 in PBS for 2 h at RT. Following two washes in TXPBS, embryos were incubated with the above-mentioned primary antibodies to detect SOX2 and CDX2, at 1:100 in 5% FCS-0.2% Tween 20 solution in PBS overnight at 4°C. After overnight incubation, embryos were washed twice in TXPBS and incubated with the appropriate secondary antibodies and DAPI in 5% FCS-0.2% Tween 20 solution in PBS for 2 h at RT and finally washed in TXPBS.

Following IHC, embryos were mounted on PBS-1% BSA microdrops made by drawing circles with a PAP pen (Kisker Biotech GmbH) on a coverslide (Bermejo-Alvarez et al., 2012). Microdrops were covered by an incubation chamber (Sigma-Aldrich, Z359467) to prevent embryo crushing. Embryos were imaged using structured illumination equipment composed of a Zeiss Axio Observer microscope coupled to an ApoTome.2 device (Zeiss). Following image acquisition, embryo diameter was measured to determine embryo size and lineage development was analysed. Total, CDX2^+^ and SOX2^+^ cell numbers were manually counted in D8 blastocysts using ZEN 2.6 software (Zeiss). In D12 embryos, hypoblast migration was determined by extension of the SOX17^+^ hypoblast layer through the inner surface of the CDX2^+^ trophectoderm, and epiblast survival was identified by the presence of SOX2^+^ cells in the embryo, whereas ED-like formation was identified by the presence of a compact structure containing SOX2^+^ cells. Total, GATA3^+^ and SOX2^+^ cell numbers were manually counted in D5 rabbit blastocysts using ZEN 2.6 software (Zeiss).

### Embryo genotyping

Embryo genotyping was performed following fixation and image analysis. Specimens (whole embryos or a fragment of E14 embryos) were placed at the bottom of a 0.2 ml PCR tube and stored at −20°C until analysis. Samples were digested with 8 (whole embryos) or 15 (E14 fragment) µl of Arcturus Picopure DNA extraction solution (Thermo Fisher Scientific) following incubation at 65°C for 1 h and inactivation at 95°C for 10 min. Lysates were subsequently used to amplify the target region by PCR as described below.

In bovine samples, the alleles composed of indels generated by NHEJ were identified by Deep Sequencing (miSeq, Illumina), as mixed peaks in Sanger chromatograms impede the identification of alleles (Lamas-Toranzo et al., 2019b). To that aim, a first PCR was performed to amplify the sequence containing the CRISPR target site adding Illumina adaptors using the primers detailed in Table S2 and adding 3 µl of lysate in a 25 µl PCR reaction (GoTaq Flexi, Promega). PCR conditions were as follows: 96°C for 2 min; 32× (96°C for 20 s, 60°C for 30 s, 72°C for 30 s); 72°C for 5 min; hold at 8°C. PCR product was purified using AMPure XP beads (Beckman Coulter^TM^) following the manufacturer's recommendations. A second 50 µl PCR was performed with the Nextera XT Index Kit v2 primers (Illumina) to add barcodes identifying each specimen, using 5 µl of each purified PCR product as a template. PCR conditions were as follows: 95°C for 3 min; 8× (95°C for 30 s, 55°C for 30 s, 72°C for 30 s); 72°C for 5 min; hold at 8°C. PCR products from different embryos were purified with AMPure XP beads, pooled into a 4 nM library and sequenced at miSeq (Illumina). Reads were aligned with the WT sequence using the BWA mem aligner (v.0.7.17; Li and Durbin, 2009), sorted and indexed with a pipeline of different tools from the SAM tools package (v.1.16.1; Li et al., 2009) and variants were called with freebayes (Garrison and Marth, 2012 preprint). VCF files were processed with an in-house script to select only potential indels within the CRISPR probe editing region and passing the quality-check filter. Results were confirmed visually checking each VCF file with the Integrative Genomics Viewer (Thorvaldsdottir et al., 2013). For representation purposes (Fig. 2C), clonal sequencing was performed as described by Lamas-Toranzo et al. (2019b). Following genotyping, embryos were classified as WT (containing no mutated alleles, i.e. all embryos in C group), IF (edited embryos containing at least one non-frame disrupting indel) and KO (containing only alleles formed by indels non-multiple of three, i.e. frame-disrupting indels).

Rabbit embryos, whose alleles were generated by cytosine base editor, were genotyped by conventional Sanger sequencing, as in the absence of indels, alleles of an identical length are clearly identifiable in the Sanger chromatogram (Fig. 5A). A PCR was performed to amplify the sequence containing the BE3 target site using the primers detailed in Table S2 and adding 3 µl of lysate in a 25 µl PCR reaction (GoTaq Flexi, Promega). PCR conditions were as follows: 96°C for 2 min; 35× (96°C for 20 s, 60°C for 30 s, 72°C for 30 s); 72°C for 5 min; hold at 8°C. PCR products were purified by FavorPrep PCR purification kit (Favorgen) and Sanger sequenced. Embryos were classified as WT (containing no mutated alleles, i.e. all embryos in the control group), heterozygotes (containing WT and KO alleles) and KO (harbouring only alleles containing the stop codon).

### Transcriptional analysis

mRNA and DNA were extracted from individual blastocysts using Dynabeads^TM^ mRNA Purification Kit following manufacturer's instructions with minor modifications. Briefly, each embryo was lysed in 20 µl of Lysis buffer and incubated with 5 µl of mRNA-binding magnetic beads. Using a magnet, the supernatant was collected to obtain the DNA following purification with AMPure XP beads (Beckman Coulter^TM^). DNA was employed to conduct embryo genotyping as previously described. Bead–mRNA complexes were washed twice before eluting mRNA in 10 µl of water at 72°C.

Once the genotype of each blastocyst was known, libraries were prepared for five WT and four KO samples, each corresponding to an individual blastocyst using SMART-Seq v4 PLUS kit (Takara Bio). Reverse transcription and PCR pre-amplification (11 cycles) were carried out with SMARTScribe II Reverse Transcriptase and SeqAmp DNA polymerase, respectively. The amplified cDNA was purified by Ampure XP beads (at a ratio of beads:sample 1:1) following manufacturer's instructions. Following cDNA quantification (Qubit, Thermo Fisher Scientific), samples were diluted to the same concentration and were processed using SMART-Seq Library Prep Kit according to the manufacturer's recommendations (Takara Bio). Following fragmentation and PCR amplification for 15 cycles, PCR products were purified using AmpPure XP beads and libraries were analysed by Qubit and Bioanalyzer (Agilent Technologies). Final cDNA libraries were sequenced by NovaSeq 6000 (Illumina) with a read length of 2×76 bp+8 bp+8 bp obtaining between 18 and 48 million reads per sample, of which 15-40 million reads were pseudoaligned after the quality control preprocessing. Differential expression was analysed by DESeq2 software obtaining raw and adjusted *P*-values and raw and shrunken fold changes calculated by the apleglm method (Zhu et al., 2019) for all genes detected.

### Statistical analyses

Data were analysed using GraphPad Prism (GraphPad Software) and SigmaStat (Systat Software) packages. χ^2^ test was used to analyse the differences in complete hypoblast migration, epiblast survival and ED-like formation between groups. Differences in blastocyst rates were analysed by *t*-test and differences in embryo survival, embryo diameter, and cell numbers between the different genotypes were analysed by one-way ANOVA. When the normality test failed, statistical differences were analysed by non-parametric *t*-test (Mann–Whitney) or one-way ANOVA (Kruskal–Wallis test).

## Supplementary Material



10.1242/develop.202993_sup1 Supplementary information

Table S1. Differentially expressed genes for the comparison of WT vs. *TEAD4* KO D8 blastocysts at an adjusted p value <0.05 and a shrunken fold change >2.
