# Supplementary material for: The role of TEAD4 in trophectoderm commitment and development is not conserved in non-rodent mammals
Source: Development. 2024 Sep 24;151(20):dev202993. doi: 10.1242/dev.202993 (PMC11463960; doi:10.1242/dev.202993)
Supplement: Supplementary information [file develop-151-202993-s1.pdf]

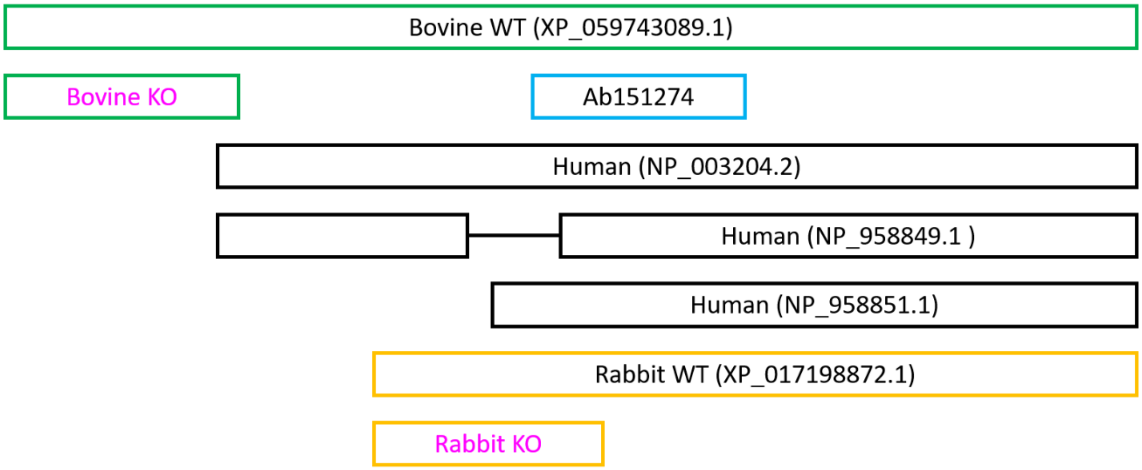


**Fig. S1. Alignment of TEAD4 WT and mutated bovine and rabbit proteins compared to human validated isoforms.** The bars length and vertical position is proportional to the aa length (the longest being XP_059743089.1, 491 aa) and alignment position following the standard N-terminal to C-terminal orientation. The frame colour of each bar indicates the species (green for bovine, black for human and orange for rabbit) and the sequence of the immunogen employed to generate the antibody Ab151274 is depicted in the blue-framed bar. Codes between brackets indicate NCBI accession numbers.

**Table S1. Differentially expressed genes for the comparison of WT vs. *TEAD4* KO D8 blastocysts at an adjusted p value <0.05 and a shrunken fold change >2.**

Available for download at
https://journals.biologists.com/dev/article-lookup/doi/10.1242/dev.202993#supplementary-data

Development • Supplementary information

**Table S2. Details of primers used**

| ID | Sequence (5´→3´) | Use |
|---|---|---|
| T7 Guide-it boTEAD4 | CCTCTAATACGACTCACTATAGGGAGAGCCCCACTCGTTGGGTTTAAGAGCTATGC | To produce gdRNA against bovine *TEAD4*. Target sequence is underlined. |
| GenoIl boTEAD4 F | TCGTCGGCAGCGTCAGATGTGTATAAGAGACAGTCTTGCCTTCATCCGACAGG | To genotype bovine embryos by miSeq. Illumina overhangs are underlined. |
| GenoIl boTEAD4 R | GTCTCGTGGGCTCGGAGATGTGTATAAGAGACAGCATTCGTCCACTGATGCCCT | |
| T7 Guide-it raTEAD4 | CCTCTAATACGACTCACTATAGGCAGCAGCAAGACCCGGACAGTTTAAGAGCTATGC | To produce gdRNA against rabbit *TEAD4*. Target sequence is underlined. |
| Geno raTEAD F | ATGTGCCCCTCCCTCTGTAT | To genotype rabbit embryos by Sanger sequencing. |
| Geno raTEAD R | GGACAGAGCAGGGAACCAAA | |

Development • Supplementary information
