# Supplementary material for: The role of TEAD4 in trophectoderm commitment and development is not conserved in non-rodent mammals
Source: Development. 2024 Sep 24;151(20):dev202993. doi: 10.1242/dev.202993 (PMC11463960; doi:10.1242/dev.202993)
Supplement: Supplementary file 1 [file develop-151-202993_review_history.pdf]

The role of TEAD4 in trophectoderm commitment and development is not conserved in non-rodent mammals

Alba Pérez-Gómez, Leopoldo González-Brusi, Inés Flores-Borobia, Beatriz Galiano-Cogolludo, Ismael Lamas-Toranzo, Julieta G. Hamze, Adolfo Toledano-Díaz, Julián Santiago-Moreno, Priscila Ramos-Ibeas and Pablo Bermejo-Álvarez
DOI: 10.1242/dev.202993

Editor: Karen Sears

Review timeline

Original submission:	25 April 2024
Editorial decision:	6 June 2024
First revision received:	10 June 2024
Editorial decision:	25 July 2024
Second revision received:	2 August 2024
Accepted:	15 August 2024

Original submission

First decision letter

MS ID#: DEVELOP/2024/202993

MS TITLE: TEAD4 role in trophectoderm commitment and development is not conserved in non-rodent mammals

AUTHORS: Alba Perez-Gomez, Leopoldo Gonzalez-Brusi, Ines Flores-Borobia, Beatriz Galiano-Cogolludo, Ismael Lamas-Toranzo, Julieta Gabriela Hamze, Adolfo Toledano, Julian Santiago-Moreno, Priscila Ramos-Ibeas, and Pablo Bermejo-Alvarez

I have now received all the referees' reports on the above manuscript, and have reached a decision. The referees' comments are appended below, or you can access them online: please go to BenchPress and click on the 'Manuscripts with Decisions' queue in the Author Area.

As you will see, the referees express considerable interest in your work, but have some significant criticisms and recommend a substantial revision of your manuscript before we can consider publication. If you are able to revise the manuscript along the lines suggested, which may involve further experiments, I will be happy receive a revised version of the manuscript. Your revised paper will be re-reviewed by one or more of the original referees, and acceptance of your manuscript will depend on your addressing satisfactorily the reviewers' major concerns. Please also note that Development will normally permit only one round of major revision. If it would be helpful, you are welcome to contact us to discuss your revision in greater detail. Please send us a point-by-point response indicating your plans for addressing the referees' comments, and we will look over this and provide further guidance.

Please attend to all of the reviewers' comments and ensure that you clearly highlight all changes made in the revised manuscript. Please avoid using 'Tracked changes' in Word files as these are lost in PDF conversion. I should be grateful if you would also provide a point-by-point response detailing how you have dealt with the points raised by the reviewers in the 'Response to Reviewers' box. If you do not agree with any of their criticisms or suggestions please explain clearly why this is so.

© 2024. Published by The Company of Biologists under the terms of the Creative Commons Attribution License (https://creativecommons.org/licenses/by/4.0/).

Reviewer 1

Advance summary and potential significance to field

This study demonstrates that developmental processes need to be studied in a variety of models and not just the mouse. Too often granting agencies and journals hold that the mouse model is the be all and end all. It is not. This study suggests that TEAD4 does not play the same role in the bovine and rabbit as it does in the mouse.

Comments for the author

This is a well-written manuscript describing the effects of gene edited KO of TEAD gene in bovine and rabbit embryos on blastocyst development. Only minor edits are suggested for the manuscript itself, as listed below. However, the authors need to add to their description of the gene edited product. When describing the gene editing, provide a more detailed description of TEAD gene.

How many exons does the gene have? Could the shortened transcript still produce a partially active protein which is not detected by the antibody? Do they know which epitopes the TEAD antibody binds. The Abcam site describes the immunogen as being a recombinant fragment corresponding to a region within amino acids 126-416 of the Human TEAD4 protein. Is this region deleted in their studies?

Other studies (see references provided below) describe shortened TEAD4 transcripts and alternate start sites. Do these studies alter the authors' interpretation or suggest that caveats might be discussed?

Suggested edits:

Line 53 to line 60: This is rather a long sentence and would benefit from editing to shorter statements.
Line 73-34: Did you mean acting at an upstream level from other TE-specific transcription factors
Line 81 … with the earlier timeline of other…
Line 139 ….showed a similar size than WT of IF embryos… Did you mean - showed a similar size to WT and IF embryos
Line 151 remove hyphen after hypoblast)
Line 186 …..as the order Rodentia…
Line 324 to the zona pellucida (not de zona)

References:
Qi, Y., Yu, J., Han, W. et al. A splicing isoform of TEAD4 attenuates the Hippo–YAP signalling to inhibit tumour proliferation. Nat Commun 7, ncomms11840 (2016). https://doi.org/10.1038/ncomms11840
Rashidiani S, Mamo G, Farkas B, Szabadi A, Farkas B, Uszkai V, Császár A, Brandt B, Kovács K, Pap M, Rauch TA. Integrative Epigenetic and Molecular Analysis Reveals a Novel Promoter for a New Isoform of the Transcription Factor TEAD4. Int J Mol Sci. 2024 Feb 13;25(4):2223. doi: 10.3390/ijms25042223. PMID: 38396900; PMCID: PMC10888684.

First revision

Author response to reviewers' comments

We wish to thank the Editor and Reviewers for their time, constructive comments and grammar corrections, which led to an improved version of the manuscript. A detailed response to the comments of Reviewer 1 can be found below. All changes in the revised version are highlighted in yellow.

© 2024. Published by The Company of Biologists under the terms of the Creative Commons Attribution License (https://creativecommons.org/licenses/by/4.0/).

Reviewer 1 Advance Summary and Potential Significance to Field...

This study demonstrates that developmental processes need to be studied in a variety of models and not just the mouse. Too often granting agencies and journals hold that the mouse model is the be all and end all. It is not. This study suggests that TEAD4 does not play the same role in the bovine and rabbit as it does in the mouse.

We thank the Reviewer for pointing out the relevant differences existing between mammals, particularly during early embryo development.

Reviewer 1 Comments for the Author...

This is a well-written manuscript describing the effects of gene edited KO of TEAD gene in bovine and rabbit embryos on blastocyst development. Only minor edits are suggested for the manuscript itself, as listed below. However, the authors need to add to their description of the gene edited product. When describing the gene editing, provide a more detailed description of TEAD gene. How many exons does the gene have? Could the shortened transcript still produce a partially active protein which is not detected by the antibody? Do they know which epitopes the TEAD antibody binds. The Abcam site describes the immunogen as being a recombinant fragment corresponding to a region within amino acids 126-416 of the Human TEAD4 protein. Is this region deleted in their studies?
Other studies (see references provided below) describe shortened TEAD4 transcripts and alternate start sites. Do these studies alter the authors' interpretation or suggest that caveats might be discussed?

We appreciate the positive perception of the reviewer and the relevant and educated comment on the possible functionality of the gene edited products. Both bovine (XM_010827947.2) and rabbit (XM_017343383) mRNA are composed by 14 exons and we have aligned the sequences of WT and KO bovine and human proteins to all human isoforms to answer the Reviewer´s questions: 1) could the alternative functional TEAD4 human proteins (NP_958849.1 and NP_958849.1, Qi *et al*. 2016 and Rashidiani *et al*. 2014) be produced by the edited bovine and rabbit *TEAD4* transcripts? and 2) Would the antibody employed detect those alternative functional products? We have included a figure (Suppl. Fig. 1) to visualize the alignment and help when following the text included in lines 282-291 and 303-312 and exposed below.

The edited product of bovine embryos shortens both bovine isoforms (XP_059743089.1 and XP_059743088.1) to the first 110 aa, and -given that the human proteins lacks the 100 aa of the bovine N-terminus- such peptide only shares the first 10 aa of two of the human isoforms (NP_003204.2 and NP_958849.1) and display no homology with the shortest (NP_958851.1). According to the antibody manufacturer (https://abiomed.kz/product/anti-tead4-antibody-ab151274) the immunogen employed to generate the antibody corresponded to aa 150-250 of NP_003204.2, which is roughly present in all human isoforms (there is only a small mismatch on NP_958849.1). The antibody recognizes a region downstream the latest alternative start site, so it should detect all three functional human TEAD4 proteins. Such immunogen correspond to residues 250-350 of the bovine protein, which are not present in the edited product, in agreement with the lack of signal on IHC. The ORF disruption site in the bovine edited transcript is downstream the canonical start site of two of the human isoforms (NP_003204.2 and NP_958849.1) and if the alternative start site of the shortest human isoform (NP_958851.1) was used by bovine embryos, the protein would have been detected by the antibody in KO embryos (as the immunogen is present in the shortest isoform).
Similarly, the edited product of rabbit embryos shortens all isoforms (XP_017198872.1, XP_051706193.1 and XP_051706192.1, sharing the same protein sequence) to 162 aa, and such edited peptide shares homology with the residues 73-235 of human NP_003204.2, the residues 73-192 of human a NP_958849.1 and the residues 1-106 of human NP_958851.1. The stop codon generated in the edited rabbit transcript is downstream the downstreamest human alternative start site (NP_958851.1), so it would also ablate the gene if such alternative start codon is in use in rabbits.

Suggested edits:

© 2024. Published by The Company of Biologists under the terms of the Creative Commons Attribution License (https://creativecommons.org/licenses/by/4.0/).

Line 53 to line 60: This is rather a long sentence and would benefit from editing to shorter statements.

Thank you for the suggestion, we have divided the sentence to improve readability (lines 55-59).

Line 73-34: Did you mean acting at an upstream level from other TE-specific transcription factors

Yes, sorry for the confusion, we have corrected it (line 73).

Line 81 … with the earlier timeline of other…

Corrected, thank you (line 81).

Line 139 ….showed a similar size than WT of IF embryos… Did you mean - showed a similar size to WT and IF embryos

Corrected, thank you (line 139).

Line 151 remove hyphen after hypoblast)

We have corrected it to ": TE and hypoblast" (lines 150-151).

Line 186 …..as the order Rodentia…

We have corrected it to ": TE and hypoblast" (lines 150-151).

Line 324 to the zona pellucida (not de zona)

Corrected, thank you (line 343).

References:
Qi, Y., Yu, J., Han, W. et al. A splicing isoform of TEAD4 attenuates the Hippo–YAP signalling to inhibit tumour proliferation. Nat Commun 7, ncomms11840 (2016). https://doi.org/10.1038/ncomms11840
Rashidiani S, Mamo G, Farkas B, Szabadi A, Farkas B, Uszkai V, Császár A, Brandt B, Kovács K, Pap M, Rauch TA. Integrative Epigenetic and Molecular Analysis Reveals a Novel Promoter for a New Isoform of the Transcription Factor TEAD4. Int J Mol Sci. 2024 Feb 13;25(4):2223. doi: 10.3390/ijms25042223. PMID: 38396900; PMCID: PMC10888684.

Inserted, as described above.

Second decision letter

MS ID#: DEVELOP/2024/202993

MS TITLE: TEAD4 role in trophectoderm commitment and development is not conserved in non-rodent mammals

AUTHORS: Alba Perez-Gomez, Leopoldo Gonzalez-Brusi, Ines Flores-Borobia, Beatriz Galiano-Cogolludo, Ismael Lamas-Toranzo, Julieta Gabriela Hamze, Adolfo Toledano, Julian Santiago-Moreno, Priscila Ramos-Ibeas, and Pablo Bermejo-Alvarez

I have now received all the referees reports on the above manuscript, and have reached a decision. The referees' comments are appended below, or you can access them online: please go to BenchPress and click on the 'Manuscripts with Decisions' queue in the Author Area.

© 2024. Published by The Company of Biologists under the terms of the Creative Commons Attribution License (https://creativecommons.org/licenses/by/4.0/).

The overall evaluation is positive and we would like to publish a revised manuscript in Development, provided that the referees' comments can be satisfactorily addressed. Please attend to all of the reviewers' comments in your revised manuscript and detail them in your point-by-point response. If you do not agree with any of their criticisms or suggestions explain clearly why this is so. If it would be helpful, you are welcome to contact us to discuss your revision in greater detail. Please send us a point-by-point response indicating your plans for addressing the referees' comments, and we will look over this and provide further guidance.

Reviewer 1

Advance summary and potential significance to field

The research reported in this manuscript clearly demonstrates that the mouse is not a good model for early embryonic development other mammals.

Comments for the author

Only minor revisions needed.

Check manuscript for extraneous hyphens.
44 layer termed zona pellucida and, around that time, a second differentiation event in the 45 ICM gives rise to the hypoblast or primitive endoderm –a second extra-embryonic 46 lineage- and the epiblast –from which the embryo proper will derive- (reviewed by 47 (Pérez-Gómez et al., 2021a)).
Lines 44-47: Check use of hyphens. Seems to be an extraneous one after 'derive'.
188 Lagomorpha-) prove that the essential role of TEAD4 appears to be exclusive for Line 188: hyphen after Lagomorpha not needed.

Revise sentences for clarity. Line 55-60: This very long run-on sentence is difficult to read and not always clear whether the statements are referring to the mouse or bovine. Moreover, how do the results in bovine contrast to the mouse if in both cases they can not form blastocysts? I believe you meant does not disrupt blastocyst formation in bovine embryos. Revise to shorter clearer sentences.
55 bovine TE (Berg et al., 2011). In the same line, whereas CDX2 is exclusively expressed 56 by the TE in mice and other mammals (Pérez-Gómez et al., 2021a), CDX2 57 downregulation (Goissis and Cibelli, 2014) or ablation ((Shi et al., 2023) and own 58 unpublished observations) in bovine embryos does disrupt blastocyst formation, which 59 contrasts to the phenotype of Cdx2 KO mice embryos, unable to maintain their 60 blastocoels due to defects in TE epithelial integrity (Strumpf et al., 2005).

Suggestion for revision.
In the same line, whereas CDX2 is exclusively expressed by the TE in mice and other mammals (Pérez-Gómez et al., 2021a), CDX2 downregulation (Goissis and Cibelli, 2014) or ablation (Shi et al., 2023; our own unpublished observations) in bovine embryos does not disrupt blastocyst formation. This contrasts with the phenotype of Cdx2 KO mice embryos, which are unable to maintain their blastocoels due to defects in TE epithelial integrity (Strumpf et al., 2005).

Line 141: Don't you mean "as D12 embryos were similar in size to WT and IF"?
141 affected by TEAD4 ablation, as D12 embryos showed a similar size than WT and IF

Second revision

Author response to reviewers' comments

We wish to thank again the Editor and Reviewer for their time and important language corrections.

Reviewer 1 Advance summary and potential significance to field

© 2024. Published by The Company of Biologists under the terms of the Creative Commons Attribution License (https://creativecommons.org/licenses/by/4.0/).

The research reported in this manuscript clearly demonstrates that the mouse is
not a good model for early embryonic development other mammals.

Reviewer 1 Comments for the author
Only minor revisions needed.

Check manuscript for extraneous hyphens.
44 layer termed zona pellucida and, around that time, a second differentiation event in the 45 ICM gives rise to the hypoblast or primitive endoderm –a second extra-embryonic 46 lineage- and the epiblast –from which the embryo proper will derive-(reviewed by 47 (Pérez-Gómez et al., 2021a)).
Lines 44-47: Check use of hyphens. Seems to be an extraneous one after 'derive'.
188 Lagomorpha-) prove that the essential role of TEAD4 appears to be exclusive for
Line 188: hyphen after Lagomorpha not needed.
We apologize for the grammar mistakes. We have changed punctuation in those lines.

Revise sentences for clarity. Line 55-60: This very long run-on sentence is difficult to read and not always clear whether the statements are referring to the mouse or bovine. Moreover, how do the results in bovine contrast to the mouse if in both cases they can not form blastocysts? I believe you meant does not disrupt blastocyst formation in bovine embryos. Revise to shorter clearer sentences.

55 bovine TE (Berg et al., 2011). In the same line, whereas CDX2 is exclusively expressed
56 by the TE in mice and other mammals (Pérez-Gómez et al., 2021a), CDX2
57 downregulation (Goissis and Cibelli, 2014) or ablation ((Shi et al., 2023) and own
58 unpublished observations) in bovine embryos does disrupt blastocyst formation, which
59 contrasts to the phenotype of Cdx2 KO mice embryos, unable to maintain their
60 blastocoels due to defects in TE epithelial integrity (Strumpf et al., 2005).

Suggestion for revision.
In the same line, whereas CDX2 is exclusively expressed by the TE in mice and other mammals (Pérez-Gómez et al., 2021a), CDX2 downregulation (Goissis and Cibelli, 2014) or ablation (Shi et al., 2023; our own unpublished observations) in bovine embryos does not disrupt blastocyst formation. This contrasts with the phenotype of Cdx2 KO mice embryos, which are unable to maintain their blastocoels due to defects in TE epithelial integrity (Strumpf et al., 2005).

We apologize for the misleading sentence. The suggestion was included and is greatly appreciated.

Line 141: Don't you mean "as D12 embryos were similar in size to WT and IF"?
141 affected by TEAD4 ablation, as D12 embryos showed a similar size than WT and IF

Yes, we have changed the sentence to "as D12 KO embryos were similar in size to WT and IF".

Third decision letter

MS ID#: DEVELOP/2024/202993

MS TITLE: TEAD4 role in trophectoderm commitment and development is not conserved in non-rodent mammals

AUTHORS: Alba Perez-Gomez, Leopoldo Gonzalez-Brusi, Ines Flores-Borobia, Beatriz Galiano-Cogolludo, Ismael Lamas-Toranzo, Julieta Gabriela Hamze, Adolfo Toledano-Diaz, Julian Santiago-Moreno, Priscila Ramos-Ibeas, and Pablo Bermejo-Alvarez
ARTICLE TYPE: Research Article

I am happy to tell you that your manuscript has been accepted for publication in Development, pending our standard publication integrity checks.

© 2024. Published by The Company of Biologists under the terms of the Creative Commons Attribution License (https://creativecommons.org/licenses/by/4.0/).
